# Effect of vitamin E supplementation on cardiometabolic risk factors, inflammatory and oxidative markers and hormonal functions in PCOS (polycystic ovary syndrome): a systematic review and meta‐analysis

**DOI:** 10.1038/s41598-022-09082-3

**Published:** 2022-04-06

**Authors:** Ghazale Tefagh, Moloud Payab, Mostafa Qorbani, Farshad Sharifi, Yasaman Sharifi, Mahbubeh Sadat Ebrahimnegad Shirvani, Farzad Pourghazi, Rasha Atlasi, Zhaleh Shadman, Nafiseh Rezaei, Erfan Mohammadi-Vajari, Bagher Larijani, Mahbube Ebrahimpur

**Affiliations:** 1 Advanced Diagnostic and Interventional Radiology Research Center, Imam Khomeini Hospital Complex, Tehran University of Medical Sciences, Tehran, Iran; 2 Endocrinology and Metabolism Research Center, Endocrinology and Metabolism Clinical Sciences Institute, Tehran University of Medical Sciences, First Floor, No 10, Jalal-Al-Ahmad Street, North Kargar Avenue, Tehran, 14117-13137 Iran; 3 Non-Communicable Diseases Research Center, Alborz University of Medical Sciences, Karaj, Iran; 4 Elderly Health Research Center, Endocrinology and Metabolism Population Sciences Institute, Tehran University of Medical Sciences, First Floor, No 10, Jalal-Al-Ahmad Street, North Kargar Avenue, Tehran, 14117-13137 Iran; 5 Department of Emergency Medicine, School of Medicine, Iran University of Medical Sciences, Tehran, Iran; 6 Evidence Based Practice Research Center, Endocrinology and Metabolism Research Institute, Tehran University of Medical Sciences, Tehran, Iran; 7 Department of Medical Library and Information Science, Paramedicine Faculty, Tehran University of Medical Sciences, Tehran, Iran; 8 Department of Medical Library and Information Science, Paramedicine Faculty, Hamadan University of Medical Sciences, Hamadan, Iran; 9 School of Medicine, Guilan University of Medical Sciences, Rasht, Iran

**Keywords:** Biomarkers, Endocrinology, Medical research, Risk factors

## Abstract

Polycystic ovary syndrome (PCOS) is a common endocrinopathy among reproductive-age women. Various therapeutical approaches are currently used to manage or control symptoms associated with PCOS. This systematic review intended to assess the effects of Vit E supplementation on cardiometabolic risk factors, inflammatory and oxidative markers, and hormonal functions in PCOS women based on the clinical trial's results. The databases including PubMed, Scopus, Cochrane, Web of Science, and Embase were used to find all relevant studies. The authors reviewed all relevant clinical trials via systematic evaluation of abstracts and titles. Searches were conducted on August 1, 2020. After the initial search and reading of the article's title and abstract, 353 articles were reviewed; finally, 12 articles met the inclusion criteria. Vitamin E supplementation improves lipid profile, decreases insulin and HOMA-IR levels. Furthermore, while Vitamin E supplementation decreases LH and testosterone concentrations, it increases FSH and progestrone concentrations. The following meta-analysis showed that vitamin E supplementation made statistically significant improvements in triglyceride (TG) and low-density lipoproteins (LDL) levels, meanwhile, pooled mean difference for waist circumference (WC) and HOMA-IR were also statistically significant. Supplementary regimens containing vitamin E can positively affect metabolic and hormonal parameters in women with PCOS.

## Introduction

Polycystic ovary syndrome (PCOS) is a common endocrinopathy among women in reproductive age with a variable prevalence between 4 and 8%, as defined by the NIH/NICHD criteria^1^. PCOS is a heterogeneous syndrome characterized by symptoms of hyperandrogenism (e.g. acne, hirsutism, and alopecia), anovulation (e.g. irregular menstrual cycles, oligomenorrhea, and amenorrhea), and polycystic ovarian morphology^2^. PCOS is associated with a variety of metabolic conditions, including type 2 diabetes mellitus (T2DM), hypertension, dyslipidemia, cardiovascular disease (CVD), and atherosclerosis^3–5^. Insulin resistance and hyperinsulinemia are common findings in PCOS as 44–70% of patients suffer from them^6,7^. Meanwhile, Dyslipidemia which can significantly decrease high-density lipoprotein (HDL), and increase triglyceride (TG) concentrations are certainly the most prevalent and persistent cardiovascular risk factors encountered in women with PCOS^8^.

The pathophysiology of PCOS is not clearly elaborated yet, but it might be associated with genetic factors, lifestyle, and deficiency of essential micronutrients in patients with insulin resistance and oxidative stress^9,10^. The first-line treatments of PCOS are mostly lifestyle modifications including exercise and diet alterations^11^, as imbalanced element status is an essential foundation for insulin resistance in PCOS ^12^. There is growing interest in using different combinations of dietary supplements such as magnesium and vitamin E, as their synergistic impact might help improve metabolic profiles in several diseases with metabolic abnormalities ^13–15^. Magnesium and vitamin E co-supplementation for 12 weeks could have beneficial effects on insulin metabolic parameters along with markers of cardio-metabolic risk in women with PCOS ^16^. Furthermore, Omega-3 fatty acids (FA) and vitamin E co-supplementations for 12 weeks in PCOS women are stated to have significantly improved insulin resistance indices and both total and free testosterone. Moreover, the beneficial effects on gene expression and oxidative stress biomarkers in this regimen have been reported ^17^. For instance, another study showed that it could significantly improve lipoprotein gene expression (a) and oxidized low-density lipoprotein, lipid profiles, and biomarkers of oxidative stress in patients with PCOS ^18^.

According to our search in the literature, there has not been a systematic review that has evaluated the role of vitamin E supplementation in PCOS treatment, this study aimed to assess the effects of vitamin E supplementation on cardiometabolic risk factors, inflammatory and oxidative markers, and hormonal functions in PCOS women based on the clinical trials' results.

## Methods

This study is reported using the Preferred Reporting Items for Systematic reviews and Meta-Analyses (PRISMA) guideline ^19^.

### Search strategy and data collection

All studies evaluating the effects of supplementary vitamin E regimens on cardiometabolic risk factors, inflammatory and oxidative markers, and hormonal functions in comparison to control group (placebo/no treatment) in PCOS patients have been searched and reviewed. The databases, including PubMed, Scopus, Web of Science, and Embase, were used to find all relevant studies. Also, the references of the relevant articles were explored to find other relevant articles. The search was not restricted to any specific time frame or language. Three emails with acceptable intervals (about two weeks) were sent to the corresponding authors of restricted access articles' for full texts. Searches were conducted on August 1, 2020, and reported the search strategy in Table 1 supplementary.

**Table 1:** Descriptions of the studies included in the systematic review and meta-analysis of the association between PCO and vitamin E supplementation.

No	Author , year	Country	Type of Study	Study Subject	Sample Size	Dose /duration of supplementation	Intervention type	Control Group	Mean Age	Outcome	Follow up duration	Measurement interval
1	Chen^22^ ^a^	China	RCT	PCOS	I = 105 C = 110	100 mg/day oral vitamin E /for 25 days	MT	Placebo: CC (100 mg/day for 5 days starting on day 3 of a spontaneous menstrual cycle or withdrawal bleeding) and HMG (75 IU every second day Starting from day 8)	26.88 ± 2.84	Estradiol Testosterone LH FSH PRL	Until miscarriage or delivery	_
2	Hager^24^	Austria	RCT	PCOS	I = 30 C = 30	30 mg vitamin E + 500 mg Omega-3 fatty acids + 800 μg folic acid, 70 μg selenium, 4 mg catechin, 12 mg glycyrrhizin, 30 mg Co-Q10 / 12 Weeks	CT	Placebo (200 μg folic acid)	27.7 ± 5.7	Testosterone SHBG FSH LH Estradiol BMI HOMA-IR	12 Weeks	Baseline and after 3 months
3	Jamilian^16^	Iran	RCT	PCOS	I = 30 C = 30	400 mg/ day Vitamin E + 250 mg/day Magnesium/12 Weeks	CT	Placebo (Barij Essence Pharmaceuticals, Kashan, Iran)	29.2 ± 7.2	Weight BMI FBS Ins HOMA-IR TC TG LDL HDL	12 Weeks	Baseline and after 3 months
4	Sadeghi^29^	Iran	RCT	PCOS	I = 32 C = 30	400 IU vitamin E + 2 omega-3 pills daily each containg : 180 mg of Eicosapentaenoic acid (EPA) and 120 mg of Docosahexaenoic acid (DHA) / 8 Weeks	CT	Placebo (oral paraffin)	26.67 ± 3.35	TAC CAT GSH MDA	8 Weeks	Baseline and after 2 months
5	Izad^26^	Iran	RCT	PCOS	I = 21 C = 21	400 IU vitamin E + 200 mg /daily CoQ10/8 Weeks	CT	Placebo (CoQ10 placebo + vitamin E placebo)	28.33 ± 5.52	BMI WC TG TC LDL HDL Non-HDL	8 Weeks	Baseline and after 2 months
6	Shokrpou^30^	Iran	RCT	PCOS	I = 30 C = 30	400 mg/day Vitamin E + 250 mg/day Magnesium/12 Weeks	CT	Placebo (Barij Essence Pharmaceuticals, Kashan, Iran)	27.2 ± 7.1	Weight BMI CRP MDA GSH TAC NO Testosterone SHBG	12 Weeks	Baseline and after 3 months
7	Jamilian^27^	Iran	RCT	PCOS	I = 20 C = 20	400 IU vitamin E + 1000 mg Omega-3 fatty acids/12 Weeks	CT	Placebo (paraffin)	22.3 ± 4.7	Weight BMI WC	12 Weeks	Baseline and after 3 months
8	Izadi^25^	Iran	RCT	PCOS	I = 21 C = 21	400 IU Vitamin E + 200 mg /daily CoQ10/8 Weeks	CT	Placebo (CoQ10 placebo + vitamin E placebo)	28.33 ± 5.52	Weight BMI FBS Ins HOMA-IR Testosterone Estradiol SHBG FSH LH Progesterone	8 Weeks	Baseline and after 2 months
9	Talari^31^	Iran	RCT	PCOS	I = 30 C = 30	400 IU vitamin E + 1000 mg Omega-3/ 12 Weeks	CT	Placebo (paraffin)	- (18–40)	NO CRP	12 Weeks	Baseline and after 3 months
10	Panti^28^	Nigeria	RCT	PCOS	I = 100 C = 100	15 mg vitamin E + 5000 IU vitamin A, 5 mg vitamin B1, 2 mg vitamin B6, 5 mg vitamin B12, 75 mg vitamin C, 400 IU vitamin D3, 45 mg Nicotinamide, 1000 mcg folic acid, 50 mg ferrous fumarate, 70 mg calcium phosphate, 0.1 mg Copper sulphate, 0.01 mg Manganese sulphate, 50 mg Zinc sulphate, 0.025 mg Potassium iodide, 0.5 mg Magnesium oxide /6 months	CT	Placebo (ferrous fumarate 100 mg)	28.18 ± 0.82	MDA	6 months	Baseline and after 6 months
11	Ebrahimi^23^	Iran	RCT	PCOS	I = 34 C = 34	400 IU Vitamin E + 1000 mg Omega-3 Fatty Acids/12 Weeks	CT	Placebo (placebos capsules by Barij Essence Kashan, Iran)	23.8 ± 4.6	Weight BMI FBS Ins HOMA-IR HOMA-B Testosterone—total Testosterone- free DHEAS SHBG	12 Weeks	Baseline and after 3 months
12	Rahmani^35^	Iran	RCT	PCOS	I = 34 C = 34	400 IU vitamin E + 1000 mg omega-3 fatty acids /12 Weeks	CT	Placebo (placebos Capsules by Barij Essence Kashan, Iran)	24.9 ± 5.5	Weight BMI TC TG LDL HDL MDA GSH TAC FSH LH	12 Weeks	Baseline and after 3 months

*RCT* randomized controlled trial, *PCOS* polycystic ovarian syndrome, *I* intervention, *C* control, *MT* mono therapy, *CT* combination therapy, *CC* clomiphene citrate, *HMG* human menopausal gonadotropin, *IU* international unit, *LH* luteinizing hormone, *FSH* follicular stimulating hormone, *PRL* prolactin, *CoQ10* co-enzyme Q10, *SHBG* sex hormone binding globulin, *HOMA-IR* homeostatic model assessment of insulin resistance, *TC* total cholesterol, *TG* triglyceride, *LDL* low density lipoprotein, *HDL* high density lipoprotein, *TAC* total antioxidant capacity, *CAT* catalase, *GSH* glutathione, *MDA* malondialdehyde, *WC* weight circumference, *FBS* fasting blood sugar, *Ins* insulin, *HOMA-B* homeostatic model assessment of beta cell function, *DHEAS* dehydroepiandrosterone sulfate.

^a^In this study intervention group consists of groups B and C with Vitamin E treatment during follicular and luteal phase, respectively.

### Inclusion criteria

Types of studies:

All relevant clinical trials (including double and single-blind and data from a parallel and cross-over group designed) evaluating the effects of vitamin E supplementary regimens in PCOS patients were gathered, and single-arm studies were not included in the study. Two authors (MM and GhT) independently screened all ofthe retrieved clinical trials using their titles and abstracts. Full-text of relevant articles were collected to assess their relevance according to the inclusion/exclusion criteria.

Types of participants:

The studies that evaluated the effects of vitamin E supplementation outcomes in the PCO adult population (≥ 18 years) were included in this study. In this regard, the subjects of the study contained patients with the PCOS receiving vitamin E supplementary regimens and control groups of PCOS patients receiving placebo or no treatment; we exclude those studies that have populations restricted to specific diseases or conditions.

Types of Interventions:

This systematic review study included all studies evaluating vitamin E supplementation (alone or as a part of combination therapy) in PCOS patients.

Types of outcomes:

The effects of vitamin E on the following outcomes were evaluated in PCOS patients:

1. Cardiometabolic risk factors including lipid profile (Total Cholesterol (TC), HDL, Low-Density Lipoprotein (LDL), TG), glycemic indices (Fasting Blood Sugar (FBS), hemoglobin A1c (HbA1c), Insulin (ins), Insulin Resistance (HOMA-IR)), and anthropometric measures (weight, body mass index (BMI), waist circumference (WC))
2. Biomarkers of inflammation and oxidative stress including C-reactive protein (CRP), plasma nitric oxide (NO), total antioxidant capacity (TAC), glutathione (GSH), malondialdehyde (MDA)
3. Sex hormones including free testosterone, total testosterone, sex hormone-binding globulin (SHBG), dehydroepiandrosterone (DHEAS), follicle-stimulating hormone (FSH), luteinizing hormone (LH), progesterone, estradiol

### Data extraction and quality assessment

Data were extracted independently from included trials by two authors according to a predefined data extraction sheet. The extracted data included (a) bibliographic and general information (author, title, publication year, type of study, randomization, and location), (b) participants (sample size and mean age), (c) intervention (type of intervention (single/combination therapy), dose of supplementation and duration), (d) control group (no treatment, placebo therapy), and (e) outcomes (reported outcomes, and follow-up time).

Two authors independently assessed the quality of included studies using the Cochrane Risk of Bias tool^20,21^.

### Statistical analysis and data synthesis

The effects of vitamin E supplementation on cardiometabolic risk factors, inflammatory and oxidative markers, and hormonal functions in PCOS women were assessed using the standardized mean difference (SMD). The meta-analysis of SMD was performed and the outcome was demonstrated as pooled standardized mean difference with 95% confidence interval. The fixed and random effect models were considered for analysis based on homogeneity of data (I^2^ < 50% considered as fix effect and I^2^ ≥ 50% considered as a random effect). The publication bias was assessed using Egger test and was presented schematically using the funnel plot. Because of the scarcity of data subgroup analysis was not carried out on the extracted data.

### Ethical considerations

In this study, ethical approval is not essential because used data are not subjects, and the results are discussed through peer‐reviewed publications.

## Results

### Description of included studies

**Figure 1:**
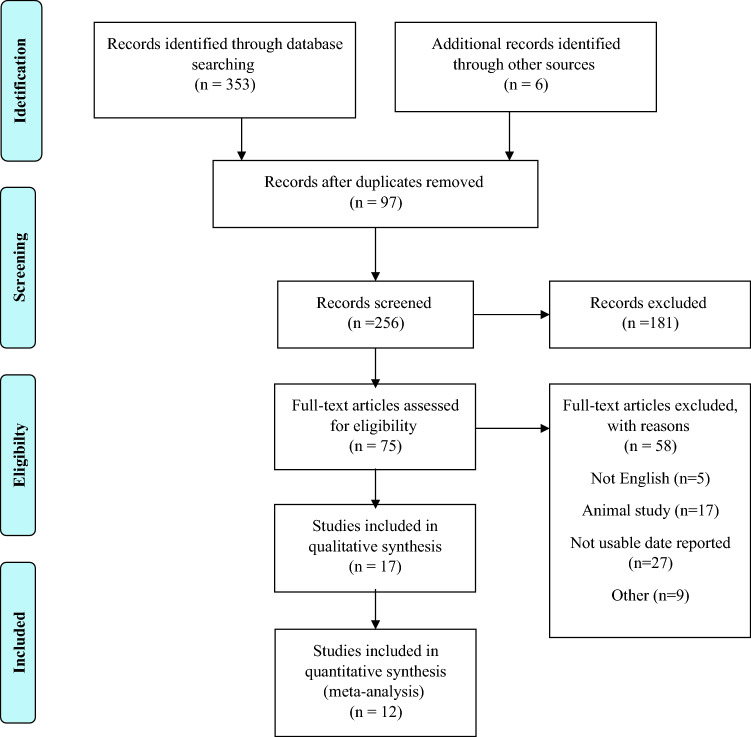
Flow chart for study identification and selection.

The flow chart of the search process and study selection is depicted in Fig. 1. Following a search on PubMed (n = 33), Scopus (n = 174), Web of Science (n = 54), and the Embase (n = 17) databases, 278 relevant articles were identified. After the initial search and reading of the article's title and abstract, 353 articles were reviewed; finally, 12 articles met the inclusion criteria^16,18,22–31^. The characteristics of included clinical trials are summarized in Table 1. Most of the studies about vitamin E and PCO treatment were conducted in Iran. Eleven studies^16,18,22–31^ evaluated the effects of vitamin E co-supplementation with other supplements such as omega 3 fatty acids and magnesium in PCOS women. Table 1 shows details different regimens used in each study.

### Quality of included studies

**Figure 2:**
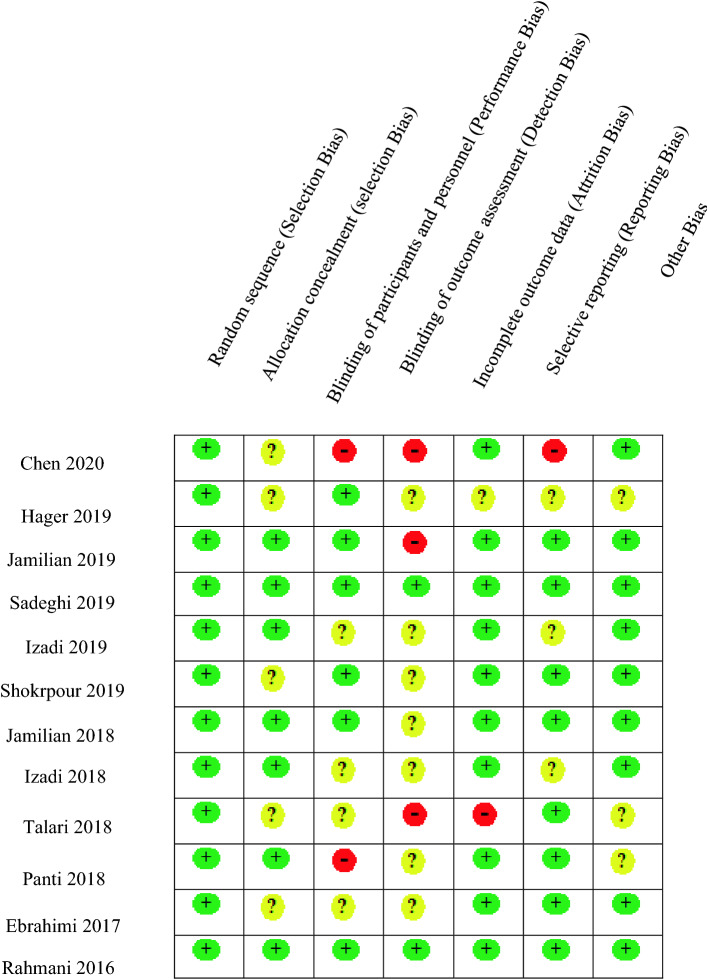
Assessment of the risk of bias in the included studies. Green circle (+): Low risk, Red circle (−): High risk, ?: Unclear.

Five studies^15,17,23,27,34^ did not describe the method used for allocation concealment clearly. Two studies^23,28^ were single-blind and four others^2,17,22,27^ did not describe the blinding process in detail. Detection bias was considered high for three studies^16,23,27^ and was unclear for nearly all other studies^2,15,17,22,24,25,28,34^. Three studies^2,22,34^ did not report some outcomes after the intervention. One study^23^ had a high risk of selective reporting bias as they did not report hormonal changes. The complete risk of bias evaluation is presented in Fig. 2. The GRADE framework^20,21^ rated the strength of the evidence for all outcomes as moderate, except for BMI^16,23,24,26,27,31–33^ and weight^16,23,24,26,27,32^, which were rated as high; progesterone^24^, LH^24,26,29,31^, FSH^24,26,29,31^, and CRP^32,34^, which were rated as low; and CAT^28^ and PRL^29^, which were rated as very low strength (Table2 supplementary) .

**Table 2:** The effect of vitamin E supplementation on cardiometabolic risk factors, inflammatory and oxidative markers, and hormonal functions in PCOS women.

Authors, year	Outcome	Intervention (mean ± SD)				Control (mean ± SD)				Between Groups		
		Before	After	Change	d	Before	After	Change	d	Change	Significance	Effect size
		Mean ± SD	Mean ± SD	Mean ± SD		Mean ± SD	Mean ± SD	Mean ± SD		Mean ± SD	NR	NR
Chen^22^ between A and B^a^	Estradiol	44.87 ± 30.52	336.51 ± 155.62	291.64 ± 139.461	− 2.60	44.47 ± 28.87	245.23 ± 126.74	200.76 ± 111.82	− 2.18	NR	NR	NR
	Testosterone	1.49 ± 0.52	NR	NR	NR	1.33 ± 0.59	NR	NR	NR	NR	NR	NR
	LH	7.44 ± 3.45	NR	NR	NR	6.94 ± 3.21	NR	NR	NR	NR	NR	NR
	FSH	5.29 ± 2.35	NR	NR	NR	5.30 ± 1.67	NR	NR	NR	NR	NR	NR
	PRL	14.97 ± 9.97	NR	NR	NR	14.24 ± 7.92	NR	NR	NR	NR	NR	NR
Chen^22^ between A and C^a^	Estradiol	45.61 ± 37.42	214.92 ± 114.11		− 1.99	44.47 ± 28.87	245.23 ± 126.74		− 2.18	NR	NR	NR
	Testosterone	1.51 ± 0.58	NR	NR	NR	1.33 ± 0.59	NR	NR	NR	NR	NR	NR
	LH	6.85 ± 2.82	NR	NR	NR	6.94 ± 3.21	NR	NR	NR	NR	NR	NR
	FSH	5.43 ± 2.44	NR	NR	NR	5.30 ± 1.67	NR	NR	NR	NR	NR	NR
	PRL	14.76 ± 8.01	NR	NR	NR	14.24 ± 7.92	NR	NR	NR	NR	NR	NR
Hager^24^	Testosterone	0.50 ± 0.19	0.43 ± 0.15	− 0.06 ± 0.09	0.40	0.43 ± 0.13	0.44 ± 0.12	0.01 ± 0.50	− 0.07	0.07 ± 0.50	NR	NR
	SHBG	46.4 ± 20.2	48.3 ± 19.2	1.8 ± 8.3	− 0.09	44.2 ± 27.3	47.1 ± 26.7	− 2.5 ± 10.6	− 0.10	− 4.30 ± 13.23	NR	NR
	FSH	5.5 ± 1.9	5.8 ± 1.8	0.4 ± 1.6	− 0.16	5.9 ± 1.6	5.2 ± 1.4	− 0.8 ± 1.9	0.46	− 1.20 ± 2.46	NR	NR
	LH	13.2 ± 6.1	10.7 ± 3.6	− 2.5 ± 4.8	0.49	11.2 ± 6.5	10.0 ± 5.3	− 1.3 ± 4.7	0.20	1.20 ± 6.67	NR	NR
	Estradiol	60.71 ± 39.60	57.18 ± 26.23	− 6.36 ± 25.65	0.10	59.61 ± 29.02	57.50 ± 23.07	− 2.11 ± 18.19	0.08	4.25 ± 31.39	NR	NR
	BMI	26.2 ± 5.6	NR	NR	NR	25.6 ± 5.4	NR	NR	NR	NR	NR	NR
	HOMA-IR	8 (26.7)	NR	NR	NR	9 (30.0)	NR	NR	NR	NR	NR	NR
Jamilian^16^	Weight	66.7 ± 9.5	66.6 ± 9.5	− 0.1 ± 0.3	0.01	67.8 ± 10.9	67.7 ± 11.1	− 0.1 ± 0.8	0.009	0 ± 0.82	NR	NR
	BMI	25.5 ± 3.5	25.5 ± 3.3	− 0.03 ± 0.1	0	26 ± 4.7	26 ± 4.7	− 0.05 ± 0.3	0	− 0.02 ± 0.27	NR	NR
	FBS	92.1 ± 12.2	90.9 ± 11.9	− 1.2 ± 6.6	0.09	93.7 ± 5.8	94.4 ± 6.5	0.7 ± 5.2	− 0.11	1.90 ± 8.36	NR	NR
	Ins	13.4 ± 5.8	12.3 ± 5.0	–1.1 ± 3.0	0.20	12.2 ± 5.1	13.9 ± 4.5	1.6 ± 3.7	− 0.35	2.70 ± 4.75	NR	NR
	HOMA-IR	3.0 ± 1.4	2.8 ± 1.2	− 0.2 ± 0.7	0.15	2.8 ± 1.2	3.2 ± 1.1	0.4 ± 0.9	− 0.34	0.60 ± 1.09	NR	NR
	TC	181.6 ± 40.4	174.5 ± 32.2	–7.0 ± 32.6	0.19	185.0 ± 34.4	193.2 ± 33.7	8.1 ± 26.6	− 0.24	15.10 ± 42.00	NR	NR
	TG	125.0 ± 53.0	110.0 ± 55.0	− 15.0 ± 24.4	0.27	128.1 ± 60.6	134.7 ± 68.9	6.7 ± 22.2	− 0.10	21.70 ± 32.92	NR	NR
	LDL	104.5 ± 36.0	101.4 ± 30.4	–3.1 ± 30.8	0.09	106.2 ± 37.1	114.2 ± 38.9	8.0 ± 27.8	− 0.21	11.10 ± 41.40	NR	NR
	HDL	52.1 ± 10.1	51.1 ± 8.6	–1.0 ± 7.0	0.10	53.1 ± 9.3	52.0 ± 10.9	–1.1 ± 6.8	0.10	− 0.10 ± 9.73	NR	NR
Sadeghi^29^	TAC	12.42 ± 1.95	13.58 ± 2.06	1.15 ± 0.93	− 0.57	12.22 ± 1.91	12.16 ± 1.96	− 0.6 ± 0.72	0.03	− 1.75 ± 0.21	NR	NR
	CAT	10.18 ± 1.27	12.01 ± 1.26	1.19 ± 1.06	− 1.44	11.14 ± 1.11	11.26 ± 1.15	0.12 ± 0.36	− 0.10	− 1.07 ± 0.20	NR	NR
	GSH	10.65 ± 2.57	12.15 ± 2.66	1.5 ± 1.06	− 0.57	10.77 ± 2.53	11.00 ± 2.65	0.23 ± 1.43	− 0.08	− 1.27 ± 0.31	NR	NR
	MDA	1.76 ± 0.29	1.42 ± 0.26	− 0.34 ± 0.32	1.23	1.38 ± 0.26	1.95 ± 2.23	0.57 ± 2.20	− 0.35	0.91 ± 0.39	NR	NR
Izadi^26^	BMI	29.28 ± 3.23	28.70 ± 3.13	− 0.59 ± 2.84	0.18	28.73 ± 3.39	28.74 ± 2.9	0.01 ± 2.84	− 0.00	0.6 ± 4.02	NR	NR
	WC	94.31 ± 8.33	91.81 ± 7.94	− 2.5 ± 7.28	0.30	89.33 ± 7.97	88.43 ± 8.04	− 0.89 ± 7.16	0.11	1.61 ± 10.2	NR	NR
	TG	108.67 ± 32.00	95.24 ± 5.86	− 13.43 ± 10.43	0.58	112.86 ± 42.27	112.09 ± 9.09	− 0.77 ± 37.52	0.02	5.51 ± 54.28	NR	NR
	TC	163.38 ± 26.30	153.86 ± 20.98	− 9.52 ± 21.67	0.40	157.43 ± 18.46	159.67 ± 22.87	2.24 ± 18.89	− 0.10	11.76 ± 28.44	NR	NR
	LDL	87.98 ± 25.64	78.67 ± 21.14	− 9.31 ± 21.30	0.39	79.57 ± 24.17	82.53 ± 22.84	2.96 ± 21.05	− 0.12	12.27 ± 29.6	NR	NR
	HDL	53.67 ± 7.44	56.14 ± 10.08	2.47 ± 8.18	− 0.27	55.28 ± 11.94	54.71 ± 9.81	− 0.57 ± 9.91	0.05	− 3.04 ± 12.64	NR	NR
	Non-HDL	109.71 ± 27.87	97.71 ± 21.72	‒11.77 (‒17.57, ‒5.97)	0.48	102.14 ± 24.39	104.95 ± 24.79	2.87 (‒2.90, 8.64)	− 0.11	NR	NR	NR
Izadi^36^ VIT E	BMI	29.28 ± 4.24	28.92 ± 4.23	− 0.363.78	_	28.73 ± 3.39	28.74 ± 2.9	0.01 ± 2.84	_	0.37 ± 4.699	NR	NR
	TC	163.41 ± 21.86	159 ± 18.96	− 4.41 ± 18.43	–	157.43 ± 18.46	159.67 ± 22.87	2.24 ± 18.89	–	6.65 ± 26.06	NR	NR
	TG	111.68 ± 44.41	105.18 ± 8.22	− 6.5 ± 40.02	–	112.86 ± 42.27	112.09 ± 9.09	− 0.77 ± 37.52	0.02	5.51 ± 54.28	NR	NR
	LDL	82.53 ± 20.51	78.1 ± 19.83	− 4.43 ± 18.05	–	79.57 ± 24.17	82.53 ± 22.84	2.96 ± 21.05	–	7.39 ± 27.36	NR	NR
	HDL	58.54 ± 9.21	59.86 ± 8.45	− 1.32 ± 7.92	–	55.28 ± 11.94	54.71 ± 9.81	− 0.57 ± 9.91	0.05	− 1.89 ± 12.51	NR	NR
	WC	95 ± 10.82	92.18 ± 10.94	− 2.82 ± 9.73	–	89.33 ± 7.92	88.43 ± 8.04	− 0.89 ± 7.16	–	1.93 ± 11.981	NR	NR
Shokrpour^30^	Weight	69.4 ± 10.7	69.2 ± 10.6	− 0.2 ± 0.3	0.01	70.9 ± 10.3	70.7 ± 10.4	− 0.1 ± 0.6	0.01	0.10 ± 0.65	NR	NR
	BMI	27.1 ± 4.2	27.0 ± 4.1	− 0.1 ± 0.1	0.02	27.9 ± 4.2	27.8 ± 4.2	− 0.1 ± 0.2	0.02	0.00 ± 0.21	NR	NR
	CRP	3.7 ± 1.9	3.1 ± 1.7	− .06 ± 1.619	0.33	3.5 ± 1.5	3.7 ± 1.5	0.2 ± 1.34	− 0.13	NR	NR	NR
	MDA	2.7 ± 0.2	2.6 ± 0.2	− 0.1 ± 0.17	0.50	2.4 ± 0.5	2.5 ± 0.5	0.1 ± 0.44	− 0.20	NR	NR	NR
	GSH	508.1 ± 69.1	519.4 ± 47.7	11.3 ± 459.62	− 0.19	481.1 ± 101.2	483.8 ± 94.2	2.7 ± 87.60	− 0.02	NR	NR	NR
	TAC	522.4 ± 30.6	590.7 ± 52.2	68.3 ± 501.52	− 1.59	513.7 ± 81.7	514.5 ± 77.3	0.8 ± 71.21	− 0.01	NR	NR	NR
	NO	34.4 ± 2.3	38.7 ± 4.0	4.3 ± 32.91	− 1.31	36.6 ± 5.6	37.0 ± 5.8	0.4 ± 5.10	− 0.07	NR	NR	NR
	Testosterone	1.4 ± 0.8	1.3 ± 0.7	− 0.1 ± 1.21	0.13	1.2 ± 0.5	1.2 ± 0.6	0 ± 0.5	0	NR	NR	NR
	SHBG	51.4 ± 26.4	62.9 ± 36.3	11.5 ± 52.14	− 0.36	48.5 ± 15.1	49.2 ± 15.2	0.7 ± 13.55	− 0.04	NR	NR	NR
Jamilian^27^	Weight	73.6 ± 11.7	72.7 ± 11.8	− 0.9 ± 1.5	0.07	69.8 ± 17.1	69.4 ± 16.9	− 0.4 ± 1.1	0.02	0.50 ± 1.83	NR	NR
	BMI	28.8 ± 5.1	28.5 ± 5.1	− 0.3 ± 0.6	0.05	26.5 ± 5.9	26.3 ± 5.8	− 0.2 ± 0.4	0.03	0.10 ± 0.71	NR	NR
	WC	90.0 ± 12.7	89.6 ± 12.6	− 0.4 ± 0.5	0.03	87.1 ± 12.4	86.9 ± 12.2	− 0.2 ± 0.6	0.01	0.20 ± 0.75	NR	NR
Izadi^25^ vit E + COQ10	Weight	75.32 ± 8.66	74.23 ± 8.9	1.43 ± 7.85	0.12	73 .23 ± 7.58	73.2 9 ± 7.3	0.15 ± 6.659	− 0.008	− 1.28 ± 10.29	NR	NR
	BMI	29.28 ± 3.23	28.7 ± 3.13	− 0.58 ± 2.84	0.25	28.7 3 ± 3.39	28.74 ± 2.9	0.01 ± 2.84	− 0.003	0.59 ± 4.021	NR	NR
	FBS	89.52 ± 18.66	81.90 ± 15.46	− 7.62 ± 15.52	0.44	79. 95 ± 9.25	80.57 ± 8.96	− 0.62 ± 8.14	− 0.06	8.24 ± 17.5	NR	NR
	Ins	15.49 ± 6.33	11.37 ± 6.44	− 4.12 ± 5.71	0.64	13.47 ± 9.73	12.47 ± 7.73	− 1 ± 8.01	0.11	3.12 ± 9.83	NR	NR
	HOMA-IR	3.30 ± 1.29	1.89 ± 0.89	− 1.41 ± 1.038	1.27	2.73 ± 2.12	2.55 ± 1.70	− 0.18 ± 1.74	0.09	1.23 ± 2.016	NR	NR
	Testosterone	1.42 ± 0.36	0.96 ± 0.32	− 0.46 ± 0.306	1.35	1.33 ± 0.35	1.47 ± 0 .39	0.14 ± 0.332	− 0.37	0.6 ± 0.45	NR	NR
	Estradiol	91.85 ± 28.16	101.65 ± 30.7	9.8 ± 26.42	− 0.33	74.43 ± 17.95	71. 09 ± 12.38	− 3.34 ± 14.44	0.21	− 13.14 ± 30.10	NR	NR
	SHBG	27.60 (21.85, 40.05)	50.30 (33.00,86.95)	NR	_	42.30 (25.20, 56.80)	40.80 (31.00, 44.50)	NR	_	NR	NR	NR
	FSH	4.60 (4.95,12.3)	6.80 (5.15,10.80)	NR	_	7.30 (3.70, 7.65)	5.90 (4.80,7.10)	NR	_	NR	NR	NR
	LH	8.50 (6.35,15.0)	7.00 (4.40,15.80)	NR	_	8.40 (5.20,17.8)	10.80 (6.70, 17.95)	NR	_	NR	NR	NR
	Progesterone	1.78 ± 0.78	2.27 ± 1.08	NR	− 0.52	1.62 ± 0.99	1.60 ± 1 .12	NR	0.01	NR	NR	NR
Izadi^25^ Vit E	Estradiol	85.45 ± 17.79	99.66 ± 23.01	14.21 ± 5.22	18.83	74.43 ± 17.95	71.09 ± 12.3	− 3.34 ± 14.4	0.21	− 17.55 ± 15.04	NR	NR
	Testosterone	1.21 ± 0.29	0.85 ± 0.21	− 0.36 ± 0.08	0.23	1.33 ± 0.35	1.47 ± 0.39	0.14 ± 0.11	− 0.37	0.5 ± 1.32	NR	NR
	HOMA-IR	2.8 ± 1.17	2.35 ± 1.01	− 0.45 ± 0.16	0.985	2.73 ± 2.12	2.55 ± 1.7	− 0.18 ± 1.74	0.09	0.27 ± 1.70	NR	NR
	FBS	85.5 ± 20.28	81.18 ± 10.28	− 4.32 ± 10	16.33	79.95 ± 9.25	80.57 ± 8.96	0.62 ± 8.14	− 0.06	4.94 ± 12.73	NR	NR
	Ins	13.72 ± 5.92	11.44 ± 4.57	12.79 ± 1.35	4.84	13.47 ± 9.73	12.47 ± 7.73	− 1 ± 8.01	0.11	− 13.79 ± 7.93	NR	NR
Talari^31^	NO	49.6 ± 2.3	51.3 ± 4.7	1.7 ± 4.7	− 0.45	46.0 ± 6.0	46.1 ± 5.9	0.1 ± 2.6	− 0.01	− 1.60 ± 5.36	NR	NR
	CRP	2877.9 ± 2095.5	2487.3 ± 1673.1	− 390.6 ± 942.9	0.20	2646.7 ± 1492.3	2883.7 ± 1488.9	237.0 ± 754.3	− 0.15	627.60 ± 1205.86	NR	NR
Panti A^28^	MDA	3.91 ± 0.05	2.89 ± 0.06	− 1.02 ± 0.05	18.46	3.99 ± 0.05	3.75 ± 1.61	− 0.24 ± 1.58	0.21	0.78 ± 1.58	NR	NR
Ebrahimi^23^	Weight	72.4 ± 10.7	71.9 ± 10.7	− 0.5 ± 1.3	0.04	75.1 ± 18.2	74.8 ± 18.3	− 0.3 ± 1.1	0.01	0.20 ± 1.69	NR	NR
	BMI	28.0 ± 4.3	27. 8 ± 4.3	− 0.2 ± 0.5	0.04	28.5 ± 6.6	28.3 ± 6.7	− 0.2 ± 0.4	0.03	0.00 ± 0.64	NR	NR
	FBS	90.2 ± 10.2	87.0 ± 8.6	− 3.2 ± 7.2	0.33	94.8 ± 7.4	94.1 ± 9.1	− 0.7 ± 6.4	0.08	2.50 ± 9.61	NR	NR
	Ins	10.8 ± 4.8	9.8 ± 4.9	− 1.0 ± 3.5	0.20	9.8 ± 5.7	12.5 ± 6.6	2.7 ± 6.6	− 0.43	3.70 ± 7.46	NR	NR
	HOMA-IR	2.4 ± 1.2	2.2 ± 1.2	− 0.2 ± 0.8	0.16	2.3 ± 1.4	2.9 ± 1.6	0.6 ± 1.5	− 0.39	0.80 ± 1.69	NR	NR
	HOMA-B	39.7 ± 18.6	35.4 ± 19.1	− 4.3 ± 14.3	0.22	33.7 ± 21.4	44.1 ± 25.4	10.5 ± 24.5	− 0.44	14.80 ± 28.33	NR	NR
	Testosterone—total	1.2 ± 0.9	0.7 ± 0.6	− 0.5 ± 0.7	0.65	1.1 ± 0.6	1.0 ± 0.6	− 0.1 ± 0.5	0.16	0.40 ± 0.81	NR	NR
	Testosterone- free	4.5 ± 3.2	3.3 ± 2.4	− 1.2 ± 2.1	0.42	3.9 ± 2.7	3.7 ± 2.3	− 0.2 ± 1.7	0.07	1.00 ± 2.68	NR	NR
	DHEAS	4.5 ± 2.3	3.5 ± 2.0	− 1.0 ± 2.1	0.46	5.2 ± 1.9	4.3 ± 1.5	− 0.9 ± 1.1	0.52	0.10 ± 2.33	NR	NR
	SHBG	37.5 ± 15.9	44.1 ± 21.3	6.6 ± 14.5	− 0.35	39.1 ± 15.0	44.9 ± 16.9	5.8 ± 13.7	− 0.36	− 0.80 ± 19.93	NR	NR
Rahmani^35^	Weight	74.1 ± 10.7	73.8 ± 10.8	− 0.3 ± 1.1	0.02	77.6 ± 18.2	77.4 ± 18.3	− 0.2 ± 1.1	0.01	0.10 ± 1.51	NR	NR
	BMI	28.4 ± 4.4	28.2 ± 4.6	− 0.1 ± 0.4	0.04	29.0 ± 6.5	29.0 ± 6.5	− 0.1 ± 0.4	0	0.00 ± 0.52	NR	NR
	TC	181.8 ± 28.0	161.5 ± 31.4	− 20.3 ± 16.6	0.68	166.4 ± 29.2	178.6 ± 29.9	12.2 ± 26.1	− 0.41	32.50 ± 30.89	NR	NR
	TG	122.7 ± 61.7	100.6 ± 54.0	− 22.1 ± 22.3	0.38	120.6 ± 59.4	128.3 ± 72.6	7.7 ± 23.6	− 0.11	29.80 ± 32.41	NR	NR
	LDL	111.1 ± 26.5	94.4 ± 29.8	− 16.7 ± 15.3	0.59	92.9 ± 25.5	104.8 ± 26.3	11.9 ± 26.1	− 0.45	28.60 ± 30.19	NR	NR
	HDL	46.2 ± 10.0	47.0 ± 9.5	0.8 ± 3.6	− 0.08	49.4 ± 8.1	48.1 ± 9.3	− 1.3 ± 6.3	0.14	− 2.10 ± 7.22	NR	NR
	MDA	2.9 ± 0.6	2.5 ± 0.6	− 0.3 ± 0.4	0.66	2.2 ± 0.5	2.2 ± 0.5	− 0.008 ± 0.6	0	0.29 ± 0.69	NR	NR
	GSH	525.3 ± 84.1	544.8 ± 81.3	19.5 ± 39.3	− 0.23	511.8 ± 69.1	555.2 ± 62.4	43.3 ± 66.3	− 0.65	23.80 ± 77.01	NR	NR
	TAC	860.5 ± 101.0	949.9 ± 119.3	89.4 ± 108.9	− 0.80	969.5 ± 85.3	975.4 ± 98.0	5.9 ± 116.2	− 0.06	− 83.50 ± 159.21	NR	NR
	FSH	7.3 ± 2.5	7.2 ± 2.5	− 0.1 ± 3.5	0.03	7.9 ± 2.8	8.1 ± 3.2	0.2 ± 3.0	− 0.06	0.30 ± 3.49	NR	NR
	LH	11.0 ± 8.0	10.5 ± 8.9	− 0.5 ± 10.1	0.05	13.5 ± 13.3	11.4 ± 7.7	− 2.1 ± 13.3	0.19	− 1.60 ± 16.67	NR	NR

^a^In this study intervention group consists of groups B and C with Vitamin E treatment during follicular and luteal phase, respectively.

*SD* standard deviation, *d* Coheoh's d, *LH* luteinizing hormone, *FSH* follicular stimulating hormone, *PRL* prolactin, *SHBG* sex hormone binding globulin, *HOMA-IR* homeostatic model assessment of insulin resistance, *FBS* fasting blood sugar, *Ins* insulin, *TC* total cholesterol, *TG* triglyceride, *LDL* low density lipoprotein, *HDL* high density lipoprotein, *TAC* total antioxidant capacity, *CAT* catalase, *GSH* glutathione, *MDA* malondialdehyde, *WC* weight circumference, *HOMA-B* homeostatic model assessment of beta cell function, *DHEAS* dehydroepiandrosterone sulfate.

## Outcomes

### Effect of vitamin E supplementation on sex hormones

**F**our studies evaluated testosterone levels pre and post Vitamin E co-supplementation (with magnesium, omega-3 FAs, and CoQ10). Table 2 shows all studies that showeda significant decrease in this regard in between the intervention group andthe control group. regarding the estradiol levels, two studies reported a similar increase in both intervention and control groups following vitamin E supplementation. In contrast, another studyreported no significant differences in estradiol levels following vitamin E + omega3 FAs supplementation. As shown on Table 2,only one study reported a small increase in estradiol levels with Vitamin E + CoQ10 supplemen group (d = -0.33) in comparison with the slight decrease that was observed in their control group (d = 0.21). Three studies evaluated Vitamin E's effect on LH levels; One study reported a medium decrease in the intervention group (d = 0.49) in comparison with the control group. This study involved simultaneous use of vitamin E, Omega-3 FA, folic acid, selenium, catechin, glycyrrhizin, and coenzyme Q10, Another study also reported a significant decrease in LH levels following vitamin E + CoQ10 supplementation. two studies reported respectively a considerable improvement in the levels of SHBG with Vitamin E + CoQ10 and vitamin E + magnesium supplementation . On the other hand, two other studies failed to show any significant change in SHBG levels following Vitamin E supplementation. Three studies that have evaluated FSH levelsand two of themshowed an increase in FSH levels with vitamin E supplementation. Meanwhile Only one study evaluated progesterone changes, and they have reported a significant increase (d = -0.52) following vitamin E + CoQ10 supplementation. Regarding DHEAS changes one study reported an increase which was not different from the increase observed in the control group. In view of the fact that gonadotropins are released in a pulsatile fashion and with various concentrations throughout the menstrual cycle and since all studies have not measured gonadotropin levels on the same point through the cycle with othersthecomparison between them is less feasible and accurate.

### Effect of vitamin E supplementation on BMI, weight

Seven studies evaluated BMI changes, but only two studies have shown significant albeit small decrease in BMI following vitamin E + CoQ10 supplementation. a study conducted in 2019 also reported a small significant decrease in waist circumference (d = 0.3). Changes in weight were not significant in either one of the studies that evaluated this concept.

### Effect of vitamin E supplementation on Insulin resistance parameters

It has been hypothesized that Vitamin E supplementation could affect insulin resistance parameters among patients with PCOS. All three studies have evaluated HOMA score and insulin level changes following dietary supplementation and have shown promising results (Table 2). one of these studies showed a significant small decrease in HOMA score and insulin level (d = 0.15 and 0.2 respectively) in their vitamin E + magnesium supplemented study group^16^. Meanwhile, another study reported a significant small decrease in HOMA-IR, HOMA-B and insulin levels (d = 0.16 and 0.22 and 0.2 respectively) following vitamin E + Omega 3 fatty acid supplementation. one of the studies reported that CoQ10 supplementation with and without vitamin E led to a significant sizeable decrease in HOMA scores and insulin levels (d = 1.27 and 0.64 respectively); however, it was also emphasized that vitamin E supplementation alone did not have a similar impact. Only one study out of these three studies,. reported a significant decrease in FBS levels.

### Effect of vitamin E supplementation on lipid profile

Vitamin E may also help PCOS patients by improving their lipid profile. Three studies that evaluated cholesterol, LDL, and TG levels changes following Vit E supplementation showed promising results. As Table 2 shows, While one of the studies reports a small significant decrease in cholesterol, LDL, and TG levels (d = 0.19 and 0.09 and 0.27 respectively) in thevitamin E + magnesium supplemented study group, anotherstudy reports a significant moderate decrease in cholesterol, LDL, and TG levels following supplementation with vitamin E + CoQ10 (d = 0.4, 0.39, and 0.58 respectively). furthermore, another study claimed a moderate to huge decrease in cholesterol, LDL, and TG levels (d = 0.68 and 0.59 and 0.38) following vitamin E + Omega 3 fatty acid supplementation. Three studies evaluated HDL levels and only one reported beneficial effects for vitamin E + CoQ10 co-supplementation.

### Effect of vitamin E supplementation on oxidative stress parameters

Some studies have suggested vitamin E supplementations may have beneficial effects on oxidation biomarkers . Vitamin E supplementation was reported to lead to a significant increase in TAC in three studies and their respective cohen’s d values is as the following: (d = -0.57), (d = -1.59) and (d = -0.8) (Table 2). One study also reported a significant increase in catalase and glutathione levels and a significant decrease in malondialdehyde levels following supplementation with vitamin E plus omega-e fatty acids (d = -1.44, -0.57, and 1.23 respectively). From the data in Table 2, the two studies suggested a significant decrease in CRP levels (d = 0.33 and 0.2 respectively) and an increase in NO levels (d = -1.3 and -0.45) after supplementation with vitamin E + magnesium and vitamin E + omega-3 fatty acids.. All three studies evaluating MDA levels reported a medium to a large decrease in values following vitamin E supplementation. Considering GSH levels, while one of the studies reported a significant small increase in the vitamin E + magnesium supplemented group (d = -0.19), another failed to show any significant change.

### Meta-analyses

#### Vitamin E and anthropometric indices

**Figure 3:**
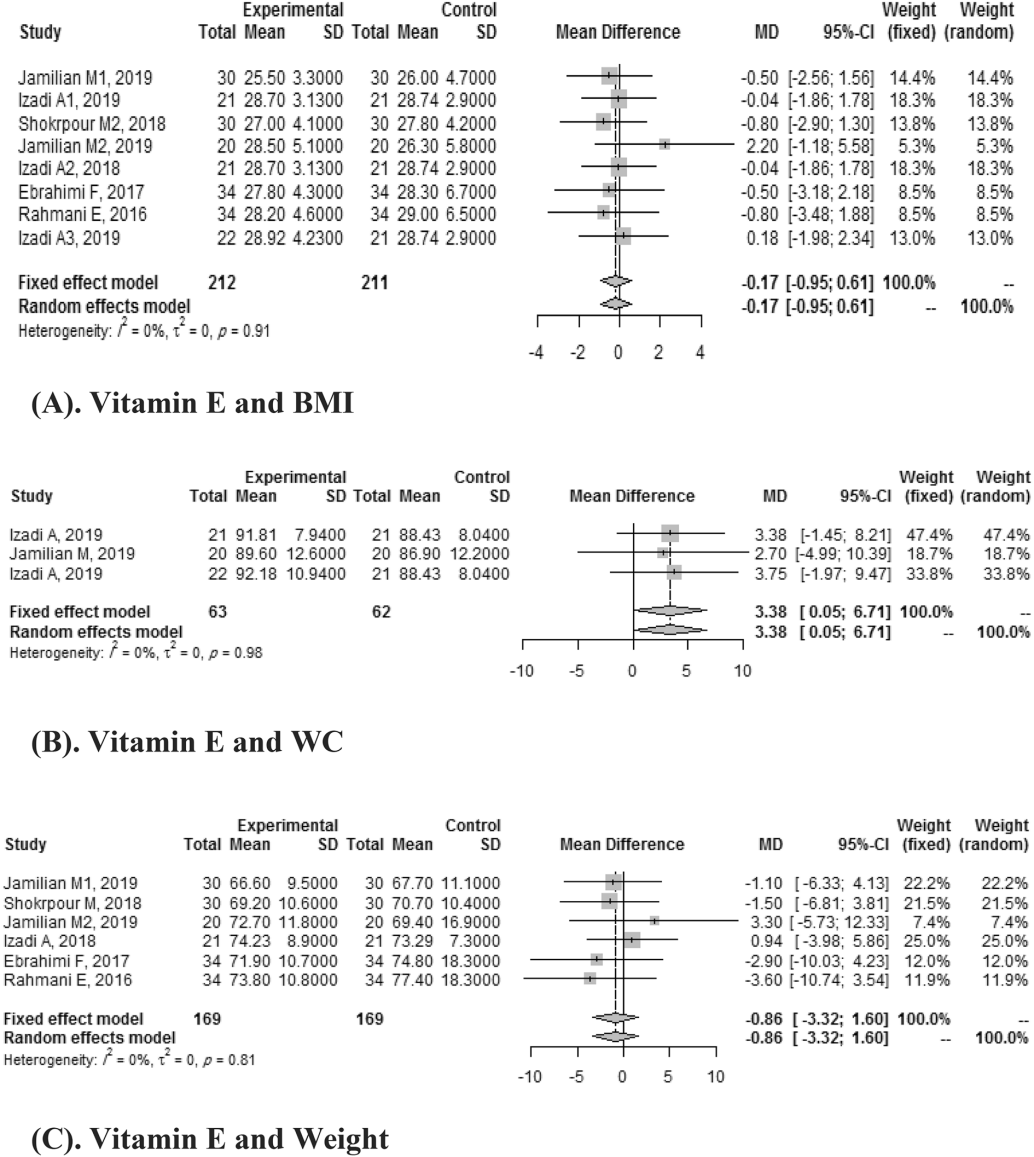
(**A**) Vitamin E and BMI. (**B**) Vitamin E and WC. (**C**) Vitamin E and weight.

Fixed effect meta-analysis of eight included studies reported the effect of vitamin E on BMI. A pooled mean difference wasn't statistically significant (SMD: -0.17, CI: 95%:-0.95, 0.61) without heterogeneity (*I* ^2^ = 0%), which means vitamin E didn't improve BMI. Three articles investigated the effect of vitamin E on WC. A pooled mean difference was found to be significant (SMD: 3.38, 95% CI: 0.05–6.71) without heterogeneity (*I* ^2^ = 0%). Six studies demonstrated the effects of Vitamin E on weight, and the pooled mean difference compared with the placebo group was -0.86 (95%CI:-3.32,1.60) without heterogeneity (*I* ^2^ = 0%) (Fig. 3).

#### Vitamin E and lipid profile

**Figure 4:**
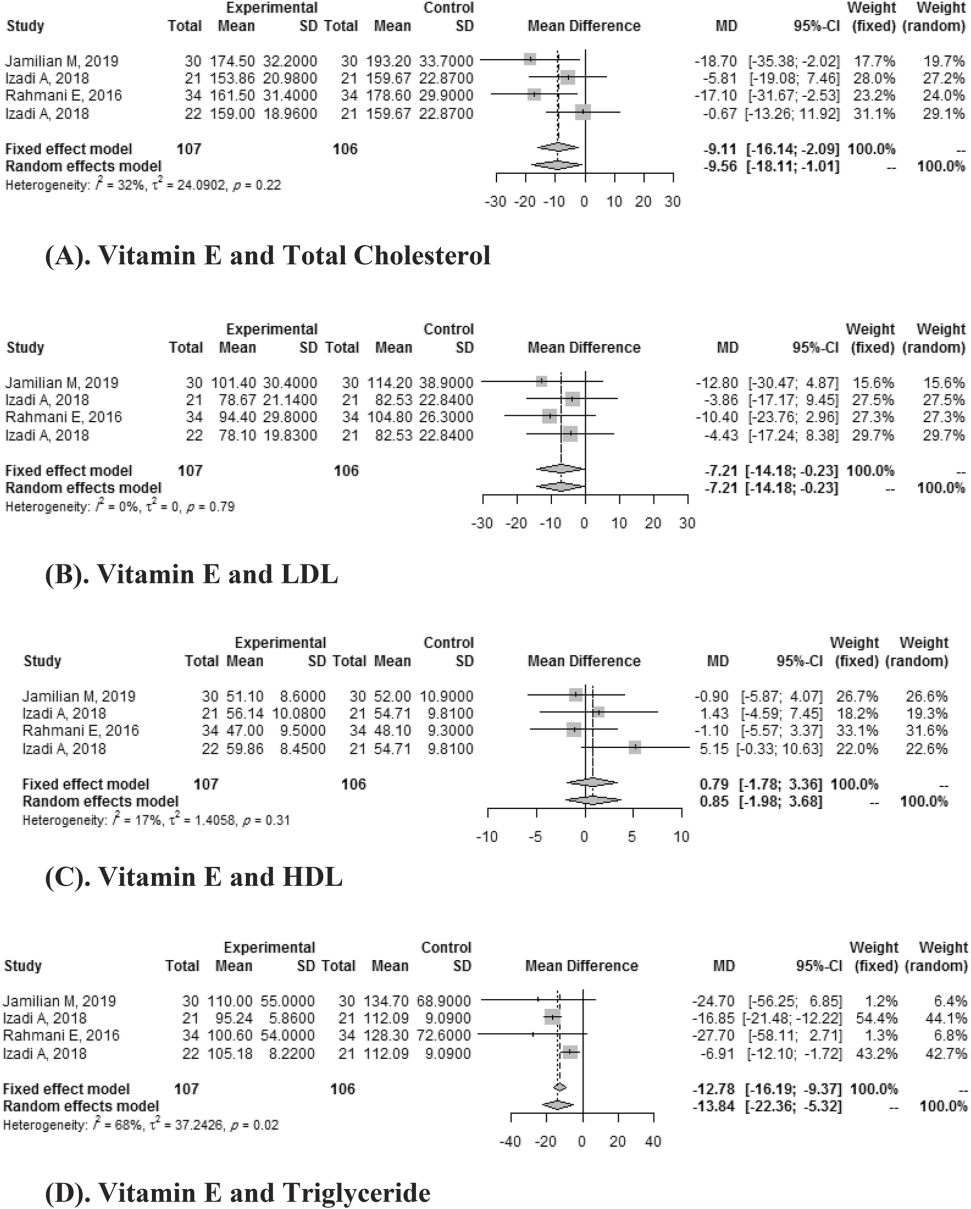
(**A**) Vitamin E and total cholesterol. (**B**). Vitamin E and LDL. (**C**) Vitamin E and HDL. (**D**) Vitamin E and triglyceride.

Four studies compared the effects of vitamin E versus placebo on TG, TC, LDL, and HDL on both baseline levels and follow-up. Overall the decrease of TC was -9.11 (95% CI: -16.14,-2.09) with 32% *I* ^2^ heterogeneities. Vitamin E did not significantly improve the HDL levels (SMD: 0.79, 95% CI: 1.78, 3.36) with *I* ^2^ heterogeneities of 17%. The meta-analyses suggested that vitamin E intake resulted in a statistically significant improvement in TG (SMD: -13.84, 95% CI:-22.36,-5.32 with *I* ^2^ heterogeneities of 68%) and LDL (SMD:-7.21, 95% CI:-14.18,-0.23 with *I* ^2^ heterogeneities of 0%) (Fig. 4).

#### Vitamin E and hormonal indices

**Figure 5:**
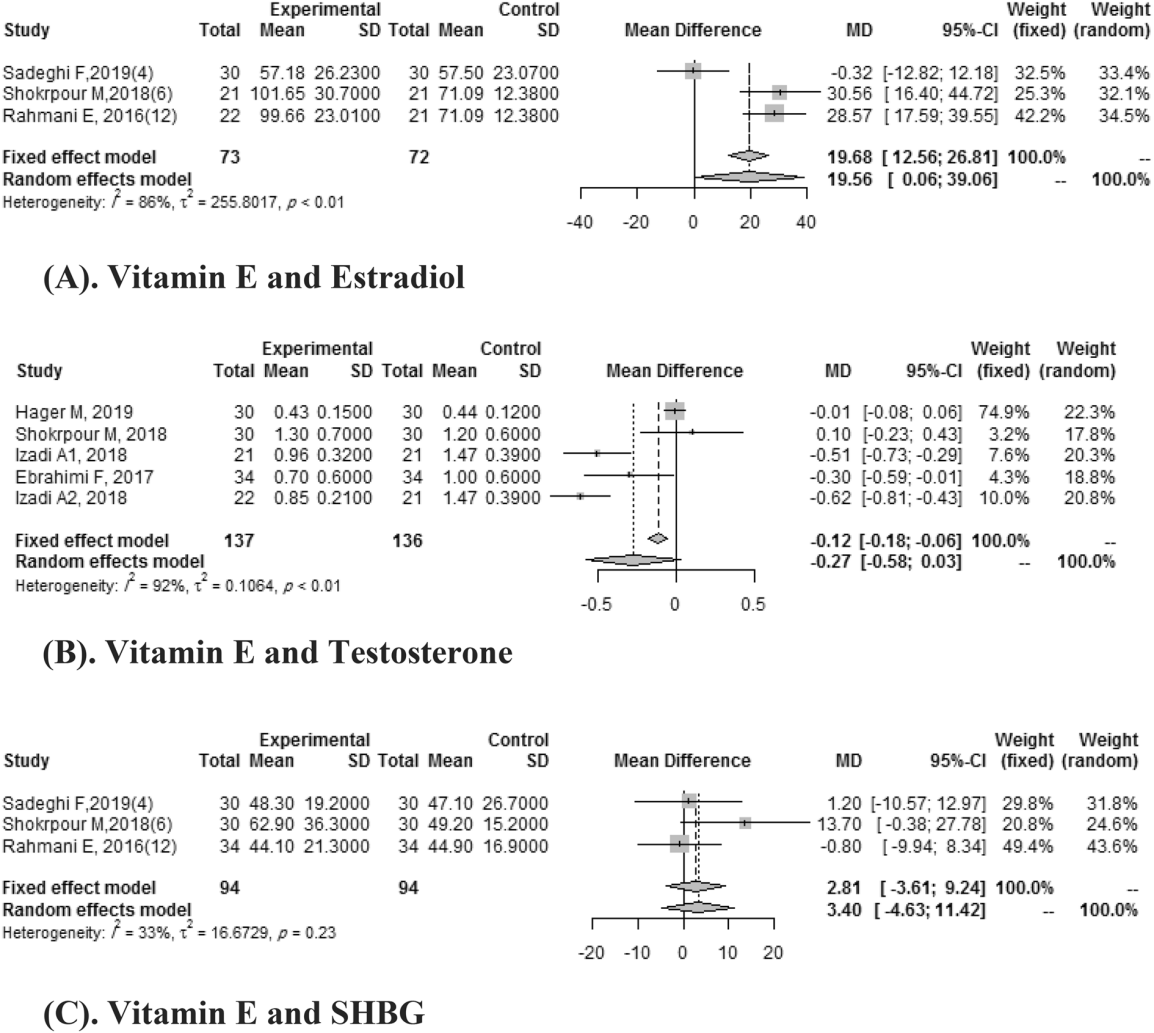
(**A**) Vitamin E and estradiol. (**B**) Vitamin E and testosterone. (**C**) Vitamin E and SHBG.

Five studies demonstrate the effects of Vitamin E intake on testosterone. A pooled mean difference wasn't significant for testosterone (SMD:-0.27, 95%CI: -0.58, 0.03) with heterogeneity (*I* ^2^ = 92%). In three studies, pooled mean difference for effects of vitamin E on estradiol compared with the placebo group was 19.56 (95% CI: 0.06, 39.06) with high heterogeneity (*I* ^2^ = 86%). Three Clinical trials reported the effect of vitamin E on SHBG. A pooled mean difference wasn't significant for SHBG (SMD: 2.81, 95%CI: -3.61, 9.24) with a heterogeneity of (I ^2^ = 32%) (Fig. 5).

#### Vitamin E and oxidation indices

**Figure 6:**
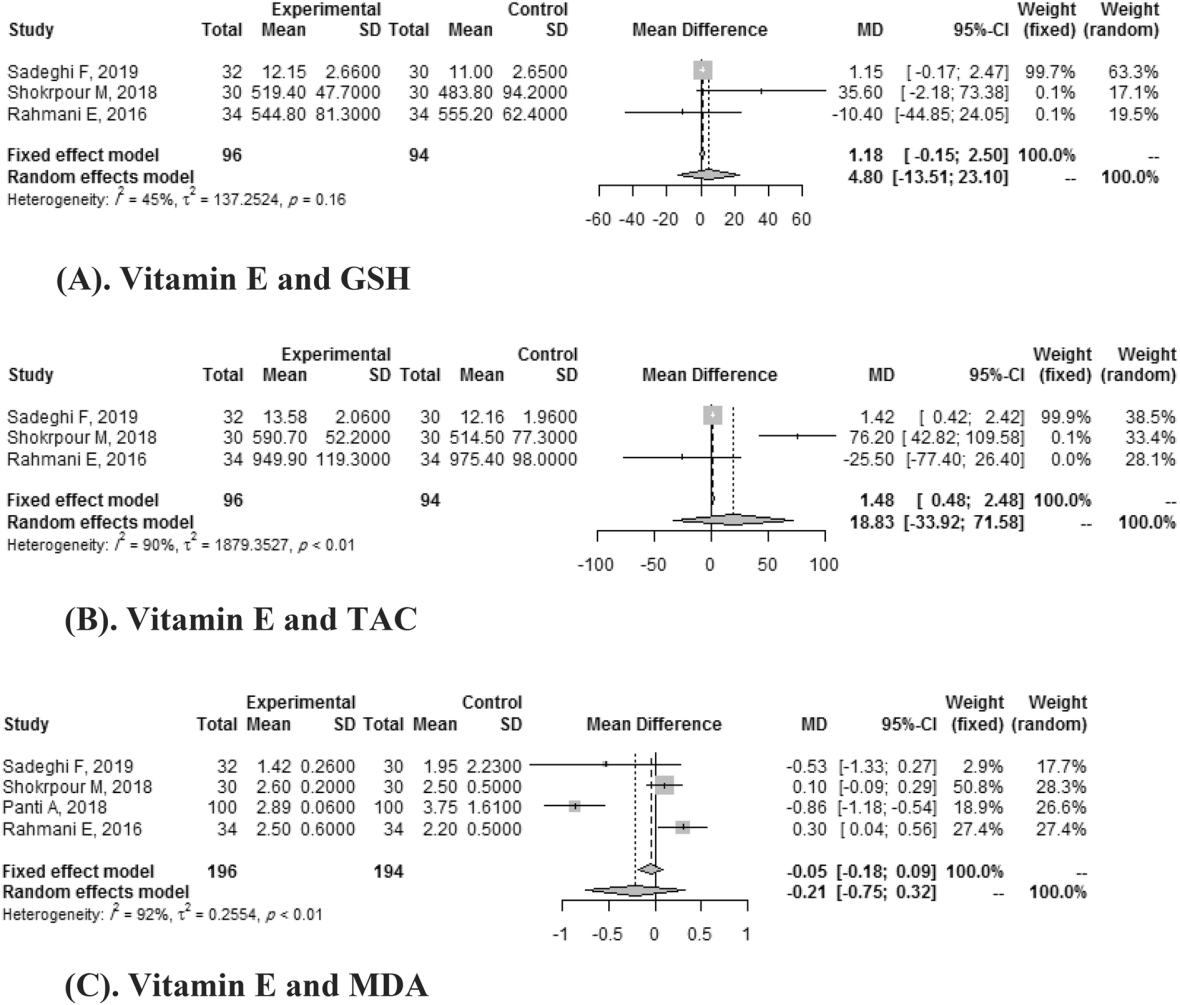
(**A**). Vitamin E and GSH. (**B**) Vitamin E and TAC. (**C**) Vitamin E and MDA.

Three clinical trials showed the effects of Vitamin E on GSH and TAC. Vitamin E didn’t significantly improve GSH 1.18(95% CI: -0.15, 2.50) with heterogeneity of (*I* ^2^ = 45%) and TAC (SMD: 18.83, 95% CI: -33.92, 2.50) with high heterogeneity (*I* ^2^ = 90%). Vitamin E didn’t significantly improve MDA either (SMD: -0.21, 95% CI:-0.75, 0.32) with high heterogeneity (*I* ^2^ = 92%) (Fig. 6).

#### Vitamin E and other indices

Four clinical trials reported the effects of vitamin E on HOMA-IR and Insulin. A pooled mean difference was significant for HOMA-IR (SMD: -0.51, CI: 95%: -0.88, -0.13) and wasn't significant for insulin (SMD: -2.82, 95% CI: -6.75, 1.11) with heterogeneity of *I* ^2^ = 52%.

**Figure 7:**
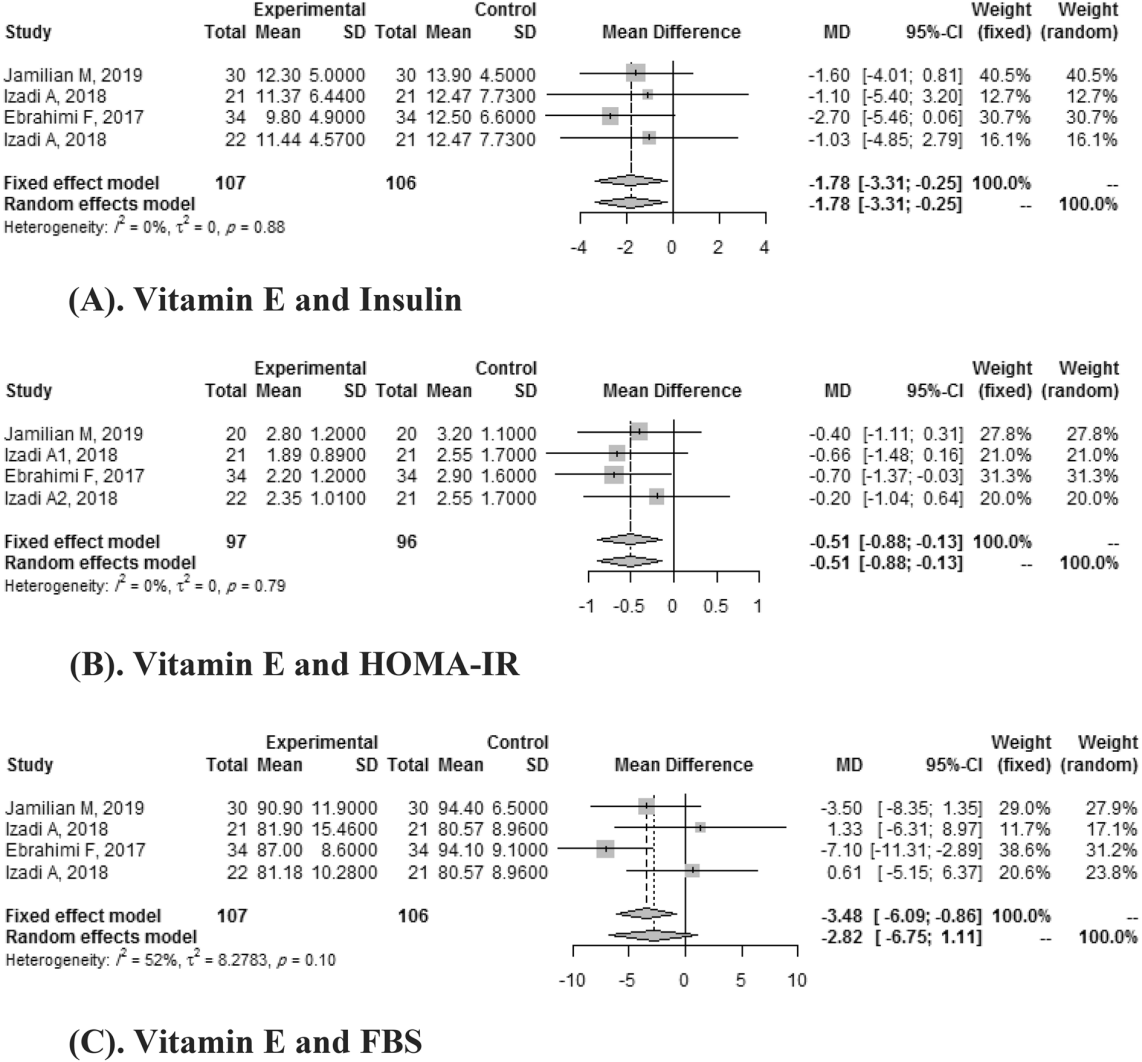
(**A**) Vitamin E and insulin. (**B**) Vitamin E and HOMA-IR. (**C**) Vitamin E and FBS.

Four articles reported the effect of vitamin E on FBS. Meta-analyses showed that vitamin E intake didn't significantly improve FBS (SMD: -2.82, 95% CI: -6.75, 1.11) with a heterogeneity of (*I* ^2^ = 52%) (Fig. 7).

## Discussion

The purpose of the current systematic review was to investigate the effects of vitamin E on cardiometabolic risk factors, inflammatory and oxidative markers, and hormonal function in PCOS patients. To our knowledge, this study is the first systematic review to assess the supplementary regimen role in PCOS treatment.

Vitamin E supplementation decreases testosterone and LH levels whereas it increases progesterone and FSH levels. So far, Studies have been unable to demonstrate a significant change in estradiol and DHEAS levels following vitamin E co-supplementation. A study by A Ciji et al. reported the effects of vitamin E supplementation to reverse oxidant agents' impact on steroid hormones such as testosterone and estradiol. To the best of our knowledge, no other review study has evaluated the effects of supplementary vitamin E regimens on steroidal hormones. No study showed a significant change in weight following vitamin E supplementationexcept for one which showed a small significant decrease in BMI following vitamin E + CoQ10 supplementation ^23^. furthermore, Insulin resistance is known to play a critical role in many PCOS comorbidities. A study conducted by Cussons AJ et al. reported that insulin resistance and obesity could lead to ventricular and endothelial dysfunction and atherosclerosis. All three studies evaluating the impact of vitamin E supplementation on insulin resistance showed decreased HOMA score and insulin levels.

A study by Renjing Xu et al. reported the beneficial effect of vitamin E on glycemic control parameters because of its antioxidant effect. And as oxidative stress might increase hemoglobin glycation ^34^. and as the detrimental effects of high blood glucose levels on pancreatic islet cells have been linked to oxidative stress. Antioxidant supplementation could manage oxidative stress.

In regards to insulin resistance and dyslipidemia, Diamanti-Kandarakis suggested that insulin resistance can increase TG and LDL levels and decrease HDL levels in PCOS patients. Moreover, they proposed that hyperandrogenism among PCOS patients may also play a role in increasing HDL levels ^33^. Vitamin E co-supplementation decreased cholesterol, LDL, and TG levels in all three studies that evaluated the effects of vitamin E supplementary regimens on lipid profile in PCOS ^2,16,35^. Sepidarkish M et al.’s study showed that vitamin E and fatty acid supplementation could only decrease VLDL levels and do not change other lipid profiles' parameters ^32^. A review and meta-analysis on the effects of omega-3 and vitamin E co-supplementation in patients with metabolic syndrome showed that this supplementary regimen could reduce both LDL and TG levels in these patients ^34^.

There is a proposed mechanism for vitamin E's beneficial effects on lipid profile improvement, lipid peroxidation ^36^ and protection of LDL from oxidation. Niki E et al. have stated that Vitamin E's anti-oxidative feature is due to its beneficial effects on oxidative stress parameters [40].

The RCTs reviewed in this study showed a significant increase in TAC, NO, catalase, glutathione, GSH levels. they have also reported a substantial decrease in malondialdehyde, CRP, and MDA levels following supplementary regimen administration in PCOS patients. A study by Sepidarkish et al. showed vitamin E, and omega-3 fatty acid co-supplementation to have increased NO levels and TAC while decreasing MDA levels ^32^.

### Strengths and limitations

This study is the first systematic review assessing the role of vitamin E supplementation in PCOS. In this systematic review, eligible studies couldn't control confusing residual variables. All of the Studies were adjusted for age and PCOS, but some of the reviews didn't consider well-defined risk factors for changing hormone levels.

This systematic review was unable to show inherent differences in vitamin E supplementation effects on PCOS between different populations and races. More studies evaluating the impact of supplementary regimens in various races and societies are needed. Moreover, due to the limited number of available studies ,we could not compare supplemental regimens' effects between different age groups. The reviewed studies have not pointed out as to whether their study populations had vitamin E deficiencies or not. Some studies have proposed that some of the beneficial effects of vitamin E supplementation might be limited to vitamin-E deficient people.

Another limitation is that due to the focus of PROSPERO (International prospective register of systematic reviews) on COVID-19 registrations during the 2020 pandemic, The PROSPERO team has not checked the eligibility of our review.

### Conclusions and implications for future research

We found that supplementary regimens containing vitamin E can positively affect the patients who are diagnosed with PCOS in regards to metabolic and hormonal parameters. It can improve their hormonal profile by decreasing testosterone and LH levels and by increasing progesterone and FSH levels. It can also reduce insulin resistance, cholesterol, LDL, and TG levels among these patients, it can also improve their cardio-metabolic profile. We also found that vitamin E supplementation can decrease oxidative stress in PCOS.

More studies are needed in order to evaluate the effects of vitamin E supplementation in different ethnicities and age groups. Other studies thatassess the effects of vitamin E supplementation in both vitamin E sufficient and deficient populations will add to current knowledge about the role of vitamin E supplementary regimens in PCOS.

## Supplementary Information

Supplementary Information.

## References

[1] Azziz R (2004). The prevalence and features of the polycystic ovary syndrome in an unselected population. J. Clin. Endocrinol. Metab..

[2] Izadi A (2019). Hormonal and metabolic effects of coenzyme Q10 and/or vitamin E in patients with polycystic ovary syndrome. J. Clin. Endocrinol. Metab..

[3] Boots, C. E. & Jungheim, E. S. Inflammation and human ovarian follicular dynamics. In *Seminars in Reproductive Medicine*, 33(4), 270–275 (2015). 10.1055/s-0035-1554928 PMC4772716 26132931

[4] Shorakae S (2019). The Emerging Role of Chronic Low-Grade Inflammation in the Pathophysiology of Polycystic Ovary Syndrome. Semin. Reprod. Med..

[5] Anagnostis P, Tarlatzis BC, Kauffman RPJM (2018). Polycystic ovarian syndrome (PCOS): Long-term metabolic consequences. Metabolism.

[6] Tsilchorozidou T, Overton C, Conway GSJCE (2004). The pathophysiology of polycystic ovary syndrome. Clin. Endocrinol. (Oxf).

[7] Jamil AS (2015). A case–control observational study of insulin resistance and metabolic syndrome among the four phenotypes of polycystic ovary syndrome based on Rotterdam criteria. Reprod. Health.

[8] Macut D, Bjekić-Macut J, Savić-Radojević A (2013). Dyslipidemia and oxidative stress in PCOS. Front. Horm. Res..

[9] Ovalle F, Azziz R (2002). Insulin resistance, polycystic ovary syndrome, and type 2 diabetes mellitus. Fertil. Steril..

[10] Zhang D (2008). The effects of oxidative stress to PCOS. Sichuan Da Xue Xue Bao Yi Xue Ban.

[11] Teede HJ (2011). Assessment and management of polycystic ovary syndrome: summary of an evidence-based guideline. Med. J. Aust..

[12] Chakraborty P (2013). Altered trace mineral milieu might play an aetiological role in the pathogenesis of polycystic ovary syndrome. Biol. Trace Elem. Res..

[13] Chang W (2014). Effects of vitamin E and magnesium on glucolipid metabolism in obese rats. Wei sheng yan jiu= Journal of hygiene research.

[14] Dou M (2009). Supplementation with magnesium and vitamin E were more effective than magnesium alone to decrease plasma lipids and blood viscosity in diabetic rats. Nutr. Res..

[15] Shokrpour, M. & Asemi, Z. J. B. T. E. R. The effects of magnesium and vitamin E co-supplementation on hormonal status and biomarkers of inflammation and oxidative stress in women with polycystic ovary syndrome. 191(1), 54–60 (2019). 10.1007/s12011-018-1602-9 30565017

[16] Jamilian M, Sabzevar NK, Asemi Z (2019). The effect of magnesium and vitamin E co-supplementation on glycemic control and markers of cardio-metabolic risk in women with polycystic ovary syndrome: A randomized, double-blind, Placebo-Controlled Trial. Hormone Metab. Res..

[17] Ebrahimi FA (2017). The effects of omega-3 fatty acids and vitamin E co-supplementation on indices of insulin resistance and hormonal parameters in patients with polycystic ovary syndrome: a randomized, double-blind, placebo-controlled trial. Hormone Metab. Res..

[18] Rahmani E (2017). The effects of omega-3 fatty acids and vitamin E co-supplementation on gene expression of lipoprotein (a) and oxidized low-density lipoprotein, lipid profiles and biomarkers of oxidative stress in patients with polycystic ovary syndrome. Mol. Cell Endocrinol..

[19] Moher D (2009). Preferred reporting items for systematic reviews and meta-analyses: The PRISMA statement (Chinese edition). PLoS Med..

[20] Guyatt GH (2008). GRADE: An emerging consensus on rating quality of evidence and strength of recommendations. BMJ.

[21] Schünemann, H., *The GRADE Handbook* (Cochrane Collaboration, 2013).

[22] Chen J (2020). Effect of a short-term vitamin E supplementation on oxidative stress in infertile PCOS women under ovulation induction: A retrospective cohort study. BMC Womens Health.

[23] Ebrahimi FA (2017). The effects of omega-3 fatty acids and vitamin E co-supplementation on indices of insulin resistance and hormonal parameters in patients with polycystic ovary syndrome: A randomized, double-blind, placebo-controlled trial. Exp. Clin. Endocrinol. Diabetes.

[24] Hager, M. *et al.* The impact of a standardized micronutrient supplementation on PCOS-typical parameters: A randomized controlled trial. **300**(2), 455–460 (2019). 10.1007/s00404-019-05194-w PMC6592962 31101977

[25] Izadi A (2018). Hormonal and metabolic effects of coenzyme Q10 and/or vitamin E in patients with polycystic ovary syndrome. J. Clin. Endocrinol. Metab..

[26] Izadi A (2019). Independent and additive effects of coenzyme Q10 and vitamin E on cardiometabolic outcomes and visceral adiposity in women with polycystic ovary syndrome. Arch. Med. Res..

[27] Jamilian M (2018). The effects of omega-3 and vitamin E co-supplementation on parameters of mental health and gene expression related to insulin and inflammation in subjects with polycystic ovary syndrome. J. Affect. Disord..

[28] Panti, A. A. *et al.* Oxidative stress and outcome of antioxidant supplementation in patients with polycystic ovarian syndrome (PCOS). **7**, 1667–1672 (2018).

[29] Sadeghi F (2019). Omega-3 and vitamin E co-supplementation can improve antioxidant markers in obese/overweight women with polycystic ovary syndrome. Int. J. Vitamin Nutr. Res..

[30] Shokrpour, M. & Asemi, Z. The effects of magnesium and vitamin E co-supplementation on hormonal status and biomarkers of inflammation and oxidative stress in women with polycystic ovary syndrome. **191**(1), 54–60 (2019). 10.1007/s12011-018-1602-9 30565017

[31] Talari HR (2018). The effects of omega-3 and vitamin e co-supplementation on carotid intima-media thickness and inflammatory factors in patients with polycystic ovary syndrome. Oman Med. J..

[32] Sepidarkish M (2020). Effect of omega-3 fatty acid plus vitamin E Co-Supplementation on oxidative stress parameters: A systematic review and meta-analysis. Clin. Nutr..

[33] Diamanti-Kandarakis E (2007). Pathophysiology and types of dyslipidemia in PCOS. Trends Endocrinol. Metab..

[34] Asbaghi O (2019). Effect of Omega-3 and vitamin E co-supplementation on serum lipids concentrations in overweight patients with metabolic disorders: A systematic review and meta-analysis of randomized controlled trials. Diabetes Metab. Syndr..

[35] Rahmani E (2017). The effects of omega-3 fatty acids and vitamin E co-supplementation on gene expression of lipoprotein (a) and oxidized low-density lipoprotein, lipid profiles and biomarkers of oxidative stress in patients with polycystic ovary syndrome. Mol. Cell. Endocrinol..

[36] Niki EJFRB (2014). Medicine, Role of vitamin E as a lipid-soluble peroxyl radical scavenger: In vitro and in vivo evidence. Free Radic Biol. Med..

