# Supplementary material for: Effect of vitamin E supplementation on cardiometabolic risk factors, inflammatory and oxidative markers and hormonal functions in PCOS (polycystic ovary syndrome): a systematic review and meta‐analysis
Source: Sci Rep. 2022 Apr 6;12:5770. doi: 10.1038/s41598-022-09082-3 (PMC8985066; doi:10.1038/s41598-022-09082-3)
Supplement: Supplementary file 1 — Supplementary Information. [file 41598_2022_9082_MOESM1_ESM.docx]

Table 1 supplementary. Search Strategy

| **PubMed**  (pcos[tiab] OR ( (polycystic[TIAB] OR cystic[TIAB] ) AND ovar*[TIAB]) OR "Polycystic Ovary Syndrome"[Mesh] OR (Sclerocystic [TIAB] AND Ovar*[TIAB])) AND ( "Vitamin E"[Mesh] OR “vit.E” [TIAB] OR tocopherol*[TIAB] OR alphatocopherol[TIAB] OR alpha-tocopherol[TIAB] OR beta-tocopherol[TIAB] OR gamma-tocopherol[TIAB] OR tocoferol*[TIAB] OR “5, 7, 8 trimethyltocol”[TIAB] OR tocopherolphosphate[TIAB] OR tocopheryl[TIAB] OR “acetic acid”[TIAB] OR “antisterility vitamin” [TIAB] OR “aquasol e” [TIAB] OR “austrovit e” [TIAB] OR covitol[TIAB] OR alphatocopherylacetate[TIAB] OR “dagravit e” [TIAB] OR dalfatol [TIAB] OR “davitamon e” [TIAB] OR dermorelle[TIAB] OR detulin[TIAB] OR “dumovit e” [TIAB] OR “e ferol” [TIAB] OR “e perle” [TIAB] OR “e perte” [TIAB] OR “e recordati” [TIAB] OR “e toplex” [TIAB] OR “e vicotrat” [TIAB] OR “e vimin” [TIAB] OR “e vita” [TIAB] OR “e viterbin” [TIAB] OR ecoferol[TIAB] OR efer[TIAB] OR eferol[TIAB] OR “enoulan forte” [TIAB] OR ephynal[TIAB] OR eplonat[TIAB] OR eprolin[TIAB] OR “epsilan m” [TIAB] OR “epsylan m” [TIAB] OR erevit[TIAB] OR esol[TIAB] OR esorb[TIAB] OR eterapion[TIAB] OR eviabit[TIAB] OR evigen[TIAB] OR eviol[TIAB] OR evion[TIAB] OR evit[TIAB] OR evitol[TIAB] OR “godabion e” [TIAB] OR gonavit[TIAB] OR hanobak[TIAB] OR “ido e” [TIAB] OR juvela[TIAB] OR juvele[TIAB] OR livingpherol [TIAB] OR “mulsal e” [TIAB] OR natopherol[TIAB] OR “optovit e” [TIAB] OR phytoferol[TIAB] OR pletocol[TIAB] OR socopherol[TIAB] OR spondyvit[TIAB] OR “toco 500” [TIAB] OR tocoferolo[TIAB] OR tocomine[TIAB] OR tocopherex[TIAB] OR tocophrine[TIAB] OR tocovigor[TIAB] OR tocovital[TIAB] OR toferol[TIAB] OR topherol[TIAB] OR “vi dom e” [TIAB] OR “vi e caps” [TIAB] OR “vi ea” [TIAB] OR “vi etal” [TIAB] OR “vibolex e” [TIAB] OR “vidom e” [TIAB] OR viea[TIAB] OR vietal[TIAB] OR viprimol[TIAB] OR “vita e” [TIAB] OR “vitamin E” [TIAB] OR viteolin[TIAB] OR viteoline [TIAB] OR “wandervit e” [TIAB] ) |
| --- |
| **WEB OF SCIENCE**  TS=((pcos OR ( (polycystic OR cystic) AND ovar*) OR (Sclerocystic AND Ovar*)) AND (“vit.E” OR tocopherol* OR alphatocopherol OR alpha-tocopherol OR beta-tocopherol OR gamma-tocopherol OR tocoferol* OR “5, 7, 8 trimethyltocol” OR tocopherolphosphate OR tocopheryl OR “acetic acid” OR “antisterility vitamin” OR “aquasol e” OR “austrovit e” OR covitol OR alphatocopherylacetate OR “dagravit e” OR dalfatol OR “davitamon e” OR dermorelle OR detulin OR “dumovit e” OR “e ferol” OR “e perle” OR “e perte” OR “e recordati” OR “e toplex” OR “e vicotrat” OR “e vimin” OR “e vita” OR “e viterbin” OR ecoferol OR efer OR eferol OR “enoulan forte” OR ephynal OR eplonat OR eprolin OR “epsilan m” OR “epsylan m” OR erevit OR esol OR esorb OR eterapion OR eviabit OR evigen OR eviol OR evion OR evit OR evitol OR “godabion e” OR gonavit OR hanobak OR “ido e” OR juvela OR juvele OR livingpherol OR “mulsal e” OR natopherol OR “optovit e” OR phytoferol OR pletocol OR socopherol OR spondyvit OR “toco 500” OR tocoferolo OR tocomine OR tocopherex OR tocophrine OR tocovigor OR tocovital OR toferol OR topherol OR “vi dom e” OR “vi e caps” OR “vi ea” OR “vi etal” OR “vibolex e” OR “vidom e” OR viea OR vietal OR viprimol OR “vita e” OR “vitamin E” OR viteolin OR viteoline OR “wandervit e” ) ) |
| **SCOPUS**  TITLE-ABS-KEY ( ( pcos  OR  ( (polycystic OR cystic)  AND  ovar* )  OR  ( sclerocystic  AND  ovar* ) )  AND  ( "vit.E"  OR  tocopherol*  OR  alphatocopherol  OR  alpha-tocopherol  OR  beta-tocopherol  OR  gamma-tocopherol  OR  tocoferol*  OR  "5, 7, 8 trimethyltocol"  OR  tocopherolphosphate  OR  tocopheryl  OR  "acetic acid"  OR  "antisterility vitamin"  OR  "aquasol e"  OR  "austrovit e"  OR  covitol  OR  alphatocopherylacetate  OR  "dagravit e"  OR  dalfatol  OR  "davitamon e"  OR  dermorelle  OR  detulin  OR  "dumovit e"  OR  "e ferol"  OR  "e perle"  OR  "e perte"  OR  "e recordati"  OR  "e toplex"  OR  "e vicotrat"  OR  "e vimin"  OR  "e vita"  OR  "e viterbin"  OR  ecoferol  OR  efer  OR  eferol  OR  "enoulan forte"  OR  ephynal  OR  eplonat  OR  eprolin  OR  "epsilan m"  OR  "epsylan m"  OR  erevit  OR  esol  OR  esorb  OR  eterapion  OR  eviabit  OR  evigen  OR  eviol  OR  evion  OR  evit  OR  evitol  OR  "godabion e"  OR  gonavit  OR  hanobak  OR  "ido e"  OR  juvela  OR  juvele  OR  livingpherol  OR  "mulsal e"  OR  natopherol  OR  "optovit e"  OR  phytoferol  OR  pletocol  OR  socopherol  OR  spondyvit  OR  "toco 500"  OR  tocoferolo  OR  tocomine  OR  tocopherex  OR  tocophrine  OR  tocovigor  OR  tocovital  OR  toferol  OR  topherol  OR  "vi dom e"  OR  "vi e caps"  OR  "vi ea"  OR  "vi etal"  OR  "vibolex e"  OR  "vidom e"  OR  viea  OR  vietal  OR  viprimol  OR  "vita e"  OR  "vitamin E"  OR  viteolin  OR  viteoline  OR  "wandervit e" ) ) |
| **EMBASE**  ('ovary polycystic disease'/exp OR pcos**:ab,ti,kw** OR ( (polycystic**:ab,ti,kw** OR cystic ) AND ovar***:ab,ti,kw**) OR (Sclerocystic **:ab,ti,kw** AND Ovar***:ab,ti,kw**))  (“vit.E”**:ab,ti,kw** OR tocopherol***:ab,ti,kw** OR alphatocopherol**:ab,ti,kw** OR alpha-tocopherol**:ab,ti,kw** OR beta-tocopherol**:ab,ti,kw** OR gamma-tocopherol**:ab,ti,kw** OR tocoferol***:ab,ti,kw** OR “5, 7, 8 trimethyltocol”**:ab,ti,kw** OR tocopherolphosphate**:ab,ti,kw** OR tocopheryl**:ab,ti,kw** OR “acetic acid”**:ab,ti,kw** OR “antisterility vitamin”**:ab,ti,kw** OR “aquasol e”**:ab,ti,kw** OR “austrovit e”**:ab,ti,kw** OR covitol**:ab,ti,kw** OR alphatocopherylacetate**:ab,ti,kw** OR “dagravit e”**:ab,ti,kw** OR dalfatol**:ab,ti,kw** OR “davitamon e”**:ab,ti,kw** OR dermorelle**:ab,ti,kw** OR detulin**:ab,ti,kw** OR “dumovit e”**:ab,ti,kw** OR “e ferol”**:ab,ti,kw** OR “e perle”**:ab,ti,kw** OR “e perte”**:ab,ti,kw** OR “e recordati”**:ab,ti,kw** OR “e toplex”**:ab,ti,kw** OR “e vicotrat”**:ab,ti,kw** OR “e vimin”**:ab,ti,kw** OR “e vita”**:ab,ti,kw** OR “e viterbin”**:ab,ti,kw** OR ecoferol**:ab,ti,kw** OR efer**:ab,ti,kw** OR eferol**:ab,ti,kw** OR “enoulan forte”**:ab,ti,kw** OR ephynal**:ab,ti,kw** OR eplonat**:ab,ti,kw** OR eprolin**:ab,ti,kw** OR “epsilan m”**:ab,ti,kw** OR “epsylan m”**:ab,ti,kw** OR erevit**:ab,ti,kw** OR esol**:ab,ti,kw** OR esorb**:ab,ti,kw** OR eterapion**:ab,ti,kw** OR eviabit**:ab,ti,kw** OR evigen**:ab,ti,kw** OR eviol**:ab,ti,kw** OR evion**:ab,ti,kw** OR evit**:ab,ti,kw** OR evitol**:ab,ti,kw** OR “godabion e”**:ab,ti,kw** OR gonavit**:ab,ti,kw** OR hanobak**:ab,ti,kw** OR “ido e”**:ab,ti,kw** OR juvela**:ab,ti,kw** OR juvele**:ab,ti,kw** OR livingpherol**:ab,ti,kw** OR “mulsal e”**:ab,ti,kw** OR natopherol**:ab,ti,kw** OR “optovit e”**:ab,ti,kw** OR phytoferol**:ab,ti,kw** OR pletocol**:ab,ti,kw** OR socopherol**:ab,ti,kw** OR spondyvit**:ab,ti,kw** OR “toco 500”**:ab,ti,kw** OR tocoferolo**:ab,ti,kw** OR tocomine**:ab,ti,kw** OR tocopherex**:ab,ti,kw** OR tocophrine**:ab,ti,kw** OR tocovigor**:ab,ti,kw** OR tocovital**:ab,ti,kw** OR toferol**:ab,ti,kw** OR topherol**:ab,ti,kw** OR “vi dom e”**:ab,ti,kw** OR “vi e caps”**:ab,ti,kw** OR “vi ea”**:ab,ti,kw** OR “vi etal”**:ab,ti,kw** OR “vibolex e”**:ab,ti,kw** OR “vidom e”**:ab,ti,kw** OR viea**:ab,ti,kw** OR vietal**:ab,ti,kw** OR viprimol**:ab,ti,kw** OR “vita e”**:ab,ti,kw** OR “vitamin E”**:ab,ti,kw** OR viteolin**:ab,ti,kw** OR viteoline**:ab,ti,kw** OR “wandervit e”**:ab,ti,kw)** |

**Table 2 Supplementary: GRADE evidence profile: Effect of Vitamin E Supplementation on Cardiometabolic Risk Factors, Inflammatory and Oxidative Markers and Hormonal Functions in PCOS**

| **Certainty assessment** | | | | | | | | **No of Patients** | | **Effect** | **Quality** |
| --- | --- | --- | --- | --- | --- | --- | --- | --- | --- | --- | --- |
| **Outcomes** | **No of studies** | **Study Design** | **Risk of bias** | **Inconsistency of results** | **Indirectness of evidence** | **Imprecision** | **Publication bias** | **Intervention groups** | **Control groups** | **Relative**  **(95% CI)** |  |
| Estradiol | 3 | RCT | not serious | not serious | not serious | not serious | not serious | 73/145 (50.3%) | 72/145 (49.7%) | **M.D=19.68**  (12.56 to 26.81) | **Moderate** |
| Progesterone | 1 | RCT | not serious | serious | not serious | not serious | not serious | 21/42 (50.0%) | 21/42 (50.0%) | Not estimable | **Low** |
| Testosterone | 5 | RCT | not serious | not serious | not serious | not serious | not serious | 137/273 (50.2%) | 136/273 (49.8%) | **M.D=-0.12** (-0.18 to -0.06) | **Moderate** |
| LH | 4 | RCT | serious | not serious | not serious | not serious | not serious | Not estimable | | | **Low** |
| FSH | 4 | RCT | serious | not serious | not serious | not serious | not serious | Not estimable | | | **Low** |
| PRL | 1 | RCT | serious | not serious | not serious | serious | not serious | Not estimable | | | **Very Low** |
| SHBG | 3 | RCT | not serious | not serious | not serious | not serious | not serious | 94/188 (50.0%) | 94/188 (50.0%) | **M.D=2.81** (-3.61 to 9.24) | **Moderate** |
| BMI | 8 | RCT | not serious | not serious | not serious | not serious | not serious | 212/423 (50.1%) | 211/423 (49.9%) | **M.D= -0.17** (-0.95 to 0.61) | **High** |
| Weight | 6 | RCT | not serious | not serious | not serious | not serious | not serious | 169/338 (50.0%) | 169/338 (50.0%) | **M.D= -0.86** (-3.32 to 1.60) | **High** |
| WC | 3 | RCT | not serious | not serious | not serious | not serious | not serious | 63/125 (50.4%) | 62/125 (49.6%) | **M.D= 3.38** (0.05 to 6.71) | **Moderate** |
| FBS | 4 | RCT | not serious | not serious | not serious | not serious | not serious | 107/213 (50.2%) | 106/213 (49.8%) | **M.D= -3.48** (-6.09 to -0.86) | **Moderate** |
| Insulin | 4 | RCT | not serious | not serious | not serious | not serious | not serious | 107/213 (50.2%) | 106/213 (49.8%) | **M.D= -1.78** (-3.31 to -0.25) | **Moderate** |
| HOMA-IR | 4 | RCT | not serious | not serious | not serious | not serious | not serious | 97/193 (50.3%) | 96/193 (49.7%) | **M.D= -0.51** (-0.88 to -0.13) | **Moderate** |
| TG | 4 | RCT | not serious | not serious | not serious | not serious | not serious | 107/213 (50.2%) | 106/213 (49.8%) | **M.D= -12.78** (-16.19 to -9.37) | **Moderate** |
| TC | 4 | RCT | not serious | not serious | not serious | not serious | not serious | 107/213 (50.2%) | 106/213 (49.8%) | **M.D= -9.11** (-16.14 to -2.09) | **Moderate** |
| LDL | 4 | RCT | not serious | not serious | not serious | not serious | not serious | 107/213 (50.2%) | 106/213 (49.8%) | **M.D= -7.21** (-14.18 to -0.23) | **Moderate** |
| HDL | 4 | RCT | not serious | not serious | not serious | not serious | not serious | 107/213 (50.2%) | 106/213 (49.8%) | **M.D= 0.79** (-1.78 to 3.36) | **Moderate** |
| CRP | 2 | RCT | serious | not serious | not serious | not serious | not serious | Not estimable | | | **Low** |
| TAC | 3 | RCT | not serious | not serious | not serious | not serious | not serious | 96/190 (50.5%) | 94/190 (49.5%) | **M.D= 1.48** (0.48 to 2.48) | **Moderate** |
| CAT | 1 | RCT | serious | not serious | not serious | serious | not serious | Not estimable | | | **Very Low** |
| GSH | 3 | RCT | not serious | not serious | not serious | not serious | not serious | 96/190 (50.5%) | 94/190 (49.5%) | **M.D= 1.18** (-0.15 to 2.50) | **Moderate** |
| MDA | 4 | RCT | not serious | not serious | not serious | not serious | not serious | 196/390 (50.3%) | 194/390 (49.7%) | **M.D= -0.05** (-0.18 to 0.09) | **Moderate** |
| CI= confidence interval, RCT= randomized control trials, MD=mean difference ,LH=luteinizing hormone, FSH=Follicle-stimulating hormone, PRL=prolactin, SHBG=sex hormone binding globulin, BMI=Body mass index, WC= Weight Circumference, FBS= Fasting Blood Sugar, HOMA-IR= Homeostatic Model Assessment of Insulin Resistance ,TG= Triglyceride ,TC= Total Cholesterol ,LDL= Low Density Lipoprotein, HDL= High Density Lipoprotein, CRP= C-reactive protein ,TAC= Total antioxidant Capacity ,CAT= Catalase ,GSH= Glutathione ,MDA= Malondialdehyde. | | | | | | | | | | | |
